# Public health roundup

**DOI:** 10.2471/BLT.19.010919

**Published:** 2019-09-01

**East African communities gear up to respond to disease outbreaks**

A field simulation exercise took place 11-14 June at the Namanga One Stop Border Post and surrounding areas in Kenya and the United Republic of Tanzania. The exercise was organized by the East Africa Community and implemented by the World Health Organization to test the two countries’ preparedness to respond to outbreaks.

**Figure:**
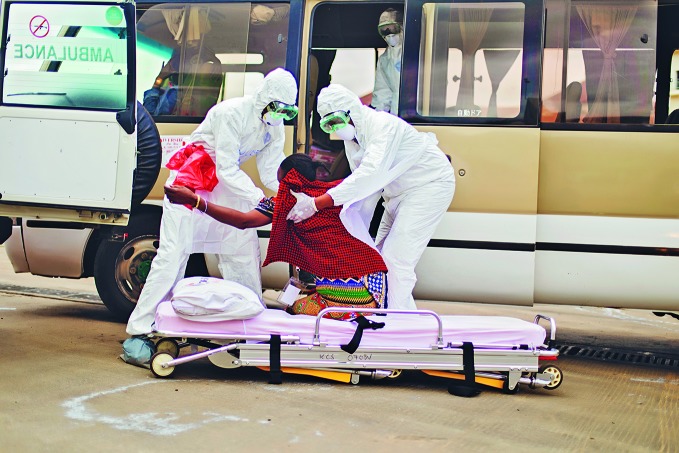
WHO/Light in Captivity

## Promising results in Ebola drug trial 

Preliminary findings in 499 study participants in a trial of four therapeutics for Ebola virus disease showed that individuals receiving REGN-EB3 or mAb114 had a greater chance of survival than participants in the other two study arms, receiving ZMapp and remdesivir.

The Pamoja Tulinde Maisha study is a randomized controlled trial of the four investigational agents for the treatment of patients with Ebola virus disease. It was launched in the Democratic Republic of the Congo in November 2018.

The preliminary findings were announced last month by the independent Data and Safety Monitoring Board that reviews interim safety and efficacy data and makes recommendations to the study team and the sponsors. 

The study is co-sponsored and funded by the Institut National de Recherche Biomédicale in Kinshasa, and the National Institute of Allergy and Infectious Diseases of the US National Institutes of Health. 

The trial is being conducted in collaboration with partners, including the Ministry of Health, the Alliance for International Medical Action, Médecins Sans Frontières, and the International Medical Corps.

As a result of an August 9, 2019 review, the Data and Safety Monitoring Board recommended that the Pamoja Tulinde Maisha study be stopped and that all future patients be randomized to receive either REGN-EB3 or mAb114 in what is being considered an extension phase of the study. 

This recommendation was based on the fact that an early stopping criterion in the protocol had been met by one of the products, REGN-EB3. 

The World Health Organization (WHO) declared the current Ebola virus disease outbreak a public health event of international concern under the International Health Regulations on 17 July and has called for more partners to join the response.

https://www.who.int/news-room/detail/11-08-2019-update-on-ebola-drug-trial-two-strong-performers-identified

## WHO recommends HIV treatment option

WHO strongly recommends the use of dolutegravir, as part of preferred first-line and second-line antiretroviral treatment for all people infected with HIV, including pregnant women and those of childbearing age.

Initial surveillance findings had highlighted a possible link between dolutegravir and neural tube defects (congenital defects of the brain and spinal cord including conditions such as spina bifida) in infants born to women using the drug at the time of conception. 

Based on those initial findings, many countries advised pregnant women and women of childbearing potential to take efavirenz instead.

However, updated data suggest that the risk of neural tube defects is lower than initially thought and there is broad consensus that the benefits of dolutegravir outweigh any risks.

Dolutegravir is more effective, easier to take and has fewer side effects than alternative HIV drugs that are currently used. 

The drug also has a high genetic barrier to developing drug resistance, which is important given the rising trend of resistance to efavirenz and nevirapine-based regimens.

https://www.who.int/news-room/detail/22-07-2019-who-recommends-dolutegravir-as-preferred-hiv-treatment-option-in-all-populations

## Tobacco control reaches more people 

Some 5 billion people are living in countries that have introduced at least one of six key tobacco control interventions at best-practice level that are proven to reduce demand for tobacco, according to a new report.

These encouraging data are reported in the seventh WHO *Report on the global tobacco epidemic, 2019*, which focuses on the progress countries have made to help tobacco users quit.

Launched in Brazil on 26 July, the report analyses national efforts to implement the most effective measures from the WHO Framework Convention on Tobacco Control (WHO FCTC) that are proven to reduce demand for tobacco. 

MPOWER stands for six demand-reduction measures: monitor tobacco use and prevention policies, protect people from tobacco smoke, offer help to quit tobacco use, warn about the dangers of tobacco, enforce bans on tobacco advertising, promotion and sponsorship and raise taxes on tobacco. 

These six MPOWER measures including smoking bans and graphic warnings on packaging were introduced in 2008 to help countries implement the demand-reduction measures of the WHO FCTC. 

Back then, the number of people protected by at least one of these measures was 1.1 billion. Since then, the figure has more than quadrupled. These six measures have been shown to save lives and reduce costs from averted health-care expenditure.

While progress is being made, with 2.4 billion people living in countries now providing comprehensive cessation services (2 billion more than in 2007), only 23 countries are providing cessation services at the best-practice level, making it the most under-implemented MPOWER measure.

https://apps.who.int/iris/bitstream/handle/10665/326043/9789241516204-eng.pdf?ua=1

## WHO calls for investment in eliminating viral hepatitis

WHO called on countries to take advantage of recent reductions in the costs of viral hepatitis treatments to scale up investments in disease elimination.

A new study by WHO, published 26 July in *Lancet Global Health*, has found that investing US$6 billion per year in eliminating hepatitis in 67 low- and middle-income countries would avert 4.5 million premature deaths by 2030, and more than 26 million deaths beyond that target date.

A total of US$ 58.7 billion is needed to eliminate viral hepatitis as a public health threat in these 67 countries by 2030. This means reducing new hepatitis infections by 90% and deaths by 65%.

“Today 80% of people living with hepatitis can’t get the services they need to prevent, test for and treat the disease,” said WHO Director-General Tedros Adhanom Ghebreyesus, calling for bold political leadership, with investments to match. 

“We call on all countries to integrate services for hepatitis into benefit packages as part of their journey towards universal health coverage,” he said.

https://www.who.int/news-room/detail/26-07-2019-who-urges-countries-to-invest-in-eliminating-hepatitis

**Cover photo**

Children receiving water provided by the Yemen Red Crescent Society in Sana'a city centre. Four years of war have resulted in severe water shortages, leaving an estimated 14.5 million people without access to safe drinking water or proper sanitation.

**Figure:**
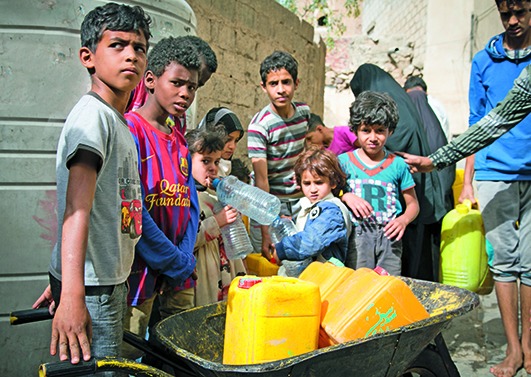
Maria Korkunc/Norwegian Red Cross

## Preventing violence through schools

Millions of children experience some form of physical, sexual or psychological violence or neglect every year, according to WHO. 

Violence in childhood can damage children’s physical and mental health and affect their whole lives, according to *School-based violence prevention: a practical handbook* that WHO published in June. 

According to data from the WHO *Global school-based student health survey,* 34% of school children reported being bullied in the previous month, while 40% reported being in a physical fight in the past year. 

Schoolchildren are also exposed to other forms of violence in educational settings, including cyber-bullying and corporal punishment, and in their homes and communities, including child maltreatment, intimate partner violence and gang violence. 

The handbook provides evidence-based guidance for teachers, school administrators, people working in education ministries and many others. 

Developed by WHO with contributions from the United Nations Children’s Fund and the United Nations Educational, Scientific and Cultural Organization, it describes in 9 strategies the steps that schools can take to implement an evidence-based approach to violence prevention. 

It provides information on how teachers can use positive discipline and thus reduce the use of corporal punishment. 

The handbook also proposes that parents strengthen their parenting skills and support children’s learning. 

The handbook can be used to implement WHO’s technical package INSPIRE: seven strategies for ending violence against children. 

https://apps.who.int/iris/bitstream/handle/10665/324930/9789241515542-eng.pdf?ua=1

## First World Patient Safety Day

The first World Patient Safety Day, on 17 September, will bring stakeholders together to reduce unintended harm caused by health care. 

The new global day will be marked by events around the world to raise awareness of patient safety, as a global health priority.

The campaign seeks to promote open communication around learning from errors and to emphasize the importance of patient safety. The slogan for World Patient Safety Day 2019 is “Speak up for patient safety!”

https://www.who.int/campaigns/world-patient-safety-day

**Looking ahead**

12 September - Global Vaccination Summit. Brussels, Belgium.

23 September - High-Level Meeting on Universal Health Coverage. New York, United States of America.

24–25 September - Sustainable Development Goals Summit. New York, United States of America.

